# Transformation through (re-)politicisation of socio-technical futures: how cultural semiotics can improve transformative vision assessment

**DOI:** 10.1186/s40309-023-00214-0

**Published:** 2023-03-21

**Authors:** Paulina Dobroć, Andreas Lösch

**Affiliations:** Institute for Technology Assessment and Systems Analysis (ITAS), Karlsruhe Institute of Technology (KIT), Postfach 3640, 76021 Karlsruhe, Germany

**Keywords:** Cultural semiotics, Transformative vision assessment, Participation processes, Technology assessment, Visions of the future, Cultural mechanism, Cultural development, Cultural techniques, 3D printing

## Abstract

The politicisation of the future is gaining attention, especially in research on the impact of emerging technologies on modern societies. This observation has motivated technology assessment (TA) and related research in science and technology studies (STS) to involve transformative practices in their examination of existing socio-technical futures in order to adapt them to societal needs. To this end, participation processes are initiated that aim to bring together different stakeholders, from research and development and beyond, to debate existing visions of the future and to confront the different stakeholders with their own ideas and the consequences thereof. Currently, however, especially in the context of responsible research and innovation (RRI), voices are also calling for reflection on the process of participation itself. We reflect on the process of framing discussions in society based on technical visions of the future from a cultural studies perspective.

Building on cultural semiotic analysis and our definition of visions of the future as cultural techniques, this paper discusses the consequences of the orientation along the future in transformative research. Cultural semiotics provides a kind of meta-reflection on the role of research in TA and STS on the politicisation of the future. We fall back on the definition of visions of the future as cultural techniques to show that visions of the future not only originate in modern culture, but also contribute to its further development.

Using the example of the transformative vision assessment project on 3D printing futures conducted within the research cluster “3D Matter Made to Order (3DMM2O)”, and based on the cultural semiotic approach, we reflect on the prerequisites and limitations of the politicisation of the future and the intervention of transformative vision assessment in politicisation processes. The limitation stems from the fact that vision assessment, and more generally TA, is oriented in its intervention towards visions of the future and thus itself contributes to the further politicisation of the future. To elaborate the preconditions of the dynamic and culture-changing effects of visions of the future, we turn to the concept of cultural mechanism to grasp different steps of the politicisation processes in which vision assessment practices are involved. The role of vision assessment in the politicisation process is unavoidable; however, it can be mitigated by meta-reflection on its own orientation to the future.

## Introduction

Politicisation of the future is increasingly discussed in technology assessment (TA), science & technology studies (STS), but also in public discourses. Means of politicising the future are—among others—technological visions of the future, i.e. ideas of the future promoted by societal actors. One of their most important characteristics is that they claim to describe fundamental social changes in the future while pointing to the need for action in the present to enable those changes here and now. The meanings ascribed to technologies in terms of their potential and future implications as “game changers” are often contested, and driven by contradictory political, economic, and other interests (e.g. [1–3].). In this way, power attributions are produced in society on the basis of future perceptions. Research can make this visible by revealing different interpretations of the discourse in the form of narratives [4]. Thus, technological visions of the future are politicised and move technologies into the position of key elements in the debates about changing societies. As a consequence, discussion in TA, STS, and related research about the purpose of technological development increases. Scientific reflection, which has the task to inform society and politics about current developments, addresses questions of how technologies can be used to meet the broadest possible societal needs. For this purpose, there is increased demand for participation processes which involve different knowledge types of various societal stakeholders, to develop research designs.

Currently, however, voices are also calling for reflection on the process of participation itself; “to question the framing assumptions [of the participation process], not just of particular policy issues” which are the subject of the participation process [5]. We pick this up and from a cultural studies perspective reflect on the process of framing discussions in society based on technical visions of the future. We will show how the approach of transformative vision assessment in the field of TA, seeks to move away from technology-centered shaping of societal development, by means of increased dialogues between different stakeholder groups involved or affected by the development of a technology. We reflect from a cultural studies perspective on how this kind of meta-reflection in transformative vision assessment can be ensured, which strives for a participatory process in the sense of RRI (responsible research and innovation). The latter claims to be a concept with a higher degree of meta-responsibility [6].

Politicisation of the future can be observed, for example, in the debates surrounding 3D printing technologies. According to their advocates, 3D printing technologies will change almost all areas of societal life. Given the growing importance of 3D printing technologies, transformative vision assessment asks to what extent they can enable cultural change, and what it actually takes for them to meet the requirements of grand societal challenges formulated in the context of RRI [7]. For this purpose, vision assessment opens the space for negotiation to broader parts of the scientific community (and in this way increases interdisciplinary dialogue), as well as to broader parts of society, in order to increase reflection on the process of shaping technologies. By opening up such dialogues, vision assessment serves as an actor in politicisation processes, as it makes the technology and its futures an issue of political negotiation in society.

The transformative vision assessment approach discussed here was conducted by a project within the *Cluster of Excellence 3D Matter Made to Order* (3DMM2O), funded by the German Research Foundation (DFG) (2019–2022).^1^ In the research cluster 3DMM2O, natural scientists and engineers are working on scalable digital 3D Additive Manufacturing. The accompanying research on 3D printing visions of the future, conducted by the project *Vision assessment of scalable 3D printing*,^2^ has shown that the hype surrounding the technology arose around 2005, when the first open source^3^ 3D printers appeared [8]. From that time, 3D printing (also called additive manufacturing) becomes the subject of public debate. The emergence of the open source 3D printer creates an opportunity to renegotiate and reinterpret the meaning of the technology. It does so by generating a *rupture* in the self-evidence of what 3D printing means and what it is being developed for. We call this *semiotic rupture*. It is a disruption in self-understanding of what a sign means, in this way it enables negotiation of the meaning and leads to politicisation processes.

The thesis of this paper is that semiotic ruptures are prerequisites of politicisation, and that the transformative vision assessment approach needs to generate such semiotic ruptures to initiate a *reflexive* politicisation process in which different parties participate in an exchange. We consider the generation of the rupture in transformative practices to be central, and at this point we see the need for operationalisable terms that describe the processes of framing and interpretation so that reflection upon them can be ensured.

Due to the range of technological applications of 3D printing technologies and the diversity of social sectors affected by them, it can be assumed that they may have a widening impact on the development of society, its values and beliefs, and thus on cultural development. As we will show, 3D printing becomes a node that brings together current societal challenges such as sustainability, inclusion, and the idea of the 4th Industrial Revolution.^4^ It leads to politicisation through negotiations of the way in which these challenges are connected to one another in the context of 3D printing. 3D printing is used as an example here to demonstrate that semiotic ruptures are necessary to achieve change, and that transformative vision assessment needs to focus on semiotic ruptures to contribute to sociotechnical change.

The main argument for turning to transformative practice in vision assessment is the need to include the knowledge of different societal actors in the process of change. But as, e.g. Stilgoe et al. [5] claim, and as we will show, reflecting on the framing process for discussion about transformation is also important, and must be considered as one of the main goals of transformative practice. It is about breaking with existing patterns of interpretation, which we believe is not done as often as is claimed. In fact, visions that circulate in society must be questioned and confronted with new contexts and perspectives. Furthermore, the orientation along technological visions of the future needs to be questionable in order to ask: What are the consequences of such framing of the negotiation process along technological visions and the future, and is it possible to achieve the cultural change that is needed by framing the process this way? The concept of *cultural mechanism*, which we will introduce in “Cultural semiotic approach to Transformative Vision Assessment”section, will clarify what causes cultural change (and at the same time how this change can be actively initiated), and how it is related to semiotic ruptures.

Due to the cultural mechanism, culture development consists of mutual influence of the culture and sign-like concepts.^5^ Accordingly, visions of the future not only contribute to cultural change, but are also a product of cultural change and thus they are interchangeable. This meta-view on visions of the future is offered by cultural studies. Drawing on the mutual influence, cultural semiotics provides the explanation of how it can be that visions of the future drive socio-technical development while they are themselves a product of this process: Because this is a reciprocal and ongoing process in which not only cultural structures influence human thoughts and actions, but also sign-like concepts can contribute to the development of culture by creating new rules. In other words: Visions of the future are not only products of social actors, but as such they also have an impact on the social actors, e.g., in defining their role in society.

In terms of observing the politicisation of the future in the context of 3D printing technologies, we pose the following research questions: (1) What is the key challenge for vision assessment towards the transformation process?; and (2) How to ensure its reflection on the process of transformation with the underlying framing assumptions?

To answer our research questions, we initially describe the role of transformative vision assessment in the development of societal visions of the future. Following the complexity of the cultural development process, we then turn to the cultural semiotic foundation of vision assessment, which situates the orientation along visions of the future and the intervention of transformative vision assessment within the process of cultural development and formation, and offers a meta-reflection on the role of visions of the future in modern culture. Finally, we interpret the interventive practices of the *Vision assessment of scalable 3D printing* project to discuss opportunities to improve the potential of the approach for cultural change.

## Transformative vision assessment in technology assessment

Transformative vision assessment is a novel application of vision assessment methodology in TA. This application intervenes explicitly in visionary discourses in science and society, with the aim to co-shape and modulate visions of future technologies. It follows the observation that visions of the future shape the scientific and societal perception of the potential and uses of an upcoming technology in the present.^6^ The aim of transformative vision assessment is to open up communicative spaces of stakeholders to anticipations that reflect how a future technology can contribute to meeting societal needs such as sustainability and social inclusion. In this way, vision assessment seeks to intervene in ongoing processes of technological development and social change by advising political, societal, and other scientific addressees regarding the impact of visions in decision-making processes. Another task is to co-productively shape the negotiated visions towards alternative or more socially robust scenarios in interaction with the relevant experts and stakeholders [17, 18]. The interactive research practices of transformative vision assessment are oriented towards the concept of “real-time technology assessment” [19] and its intervening methods of “sociotechnical integration research” [20]. The practices are oriented towards the normative frameworks of RRI (for an overview see [7]).

The central analytical interest of vision assessment is the *performativity* of visions of the future in current processes of change, caused by their *mediality*. Impacts of visions emerge as they serve as *means* *to enable* *communication* [21] in contexts of their discursive and practical use: In these contexts, visions enable translations between the present and the future, communications between contradictory perspectives, coordination between heterogeneous actors, and activate actors to contribute [22]. Transformative vision assessment strives to use analytical insights on the performativity and mediality of visions to modulate the visions in interaction with actors from science and society towards sociotechnical scenarios. Its methodological horizon combines the monitoring of visionary debates and practices with an interactive, socio-epistemic co-creation and transformation of visions. This approach aims to reflexively use visions and visionary communication to promote RRI [23] in interaction with the relevant actors. Means of the intervening modulation are, for example, the reflexive use of contradictory visionary key narratives [24], which are elaborated in the analysis process, in communicative interactions, and the reflexive co-creation of sociotechnical scenarios with scientists and other stakeholders [25].

In a transformative vision assessment research project, the approach works on three interrelated process-dimensions: (1) the analytical exploration of the visionary space of a future technology; (2) feedback-dialogues with diverse relevant actor groups, and (3) the modulation of visions of the future towards sociotechnical scenarios that embed visions of future technology in societal and ecological needs and challenges, in order to improve the reflexivity of relevant actors to make decisions and take actions [26]. In the course of this transition from the use of visionary narratives as means of communication towards the co-creation of sociotechnical scenarios, the transformative act of the vision assessment is executed. With such interactive practices of vision assessment, TA becomes an actor in the societal negotiations and itself contributes to the politicisation of the future.

Against this background, vision assessment undertakes an interpretative practice. In this paper, we assume that this activity usually addresses mostly well-known social patterns, such as interpretative patterns, to explain the transformation processes. However, our *question is*: What about the newly emerging social patterns, as well as patterns in which thinking along visions of the future is involved? Can they be captured by such interpretative practices, and if so, how? Are they captured by the transformative acts of modulating visions towards the sociotechnical scenarios? Vision assessment perceives itself as a transformative practice which enables or fosters processes of RRI. Therefore, following calls for more self-reflexivity of anticipatory and participatory processes (e.g. [5, 27]), there is a need to understand the emerging societal patterns and rules. After all, vision assessment is called upon to question the existing order and support change. In the following, we reflect on how such societal changes occur in the practices of transformative vision assessment projects, and how those practices can be improved.

As mentioned in the introduction, this project was an accompanying social science study in a research cluster working on the development of scalable 3D printing, 3DMM2O. According to the mission of transformative vision assessment, the project served to embed the research of 3DMM2O into societal discourses. In this way, by communicating along visions of the future around the development of 3D printing, dialogues with stakeholders from beyond research were included. The aim was to broaden the anticipatory space of thinking of the researchers and stakeholders by transforming the usual modes of thinking about future 3D printing *technologies as a solution* *for societal needs,* towards *societal changes as conditions* of unfolding the potential of 3D printing to contribute to urgent societal transformations. That is, to make the technologies a subject of reflexive political negotiations in society (see “On the transformative process of Vision Assessment on 3D printing futures” section).

But what has been lacking to date is self-refection on the processes of such a project, e.g. on the conditions and limitations of the transformative impact of the vision assessment approach.^7^ The following cultural semiotic approach reflects on those conditions and limitations. It shows (1) how the rulemaking process in which vision assessment intervenes fosters the politicisation of (here, 3D printing) technologies, and (2) why and how the focus on visions of the future simultaneously leads to a limitation of this intervention. This reflection on the process of the interventive practices of transformative vision assessment is a prerequisite of transformative research, which intends to realise responsible processes of innovation. As such, the approach is a self-learning enterprise in practice.

## Cultural semiotic approach to transformative vision assessment

This section introduces the basic assumptions and concepts of cultural semiotics that we use in the following reflection on the conditions and limitations of the intervention of transformative vision assessment for unfolding its transformative potential. Cultural semiotics is referred to as a sub-discipline of semiotics that deals with the role of signs in cultural development. The philosopher Ernst Cassirer claims that signs play a constitutive role in the formation of culture, and cultures are therefore performed by means of signs ([28–30], see also [31, 32]). This emphasises the double-sided character of signs such as visions of the future: Visions not only create culture but are also a product of cultural development, and as such they are contingent and to be questioned. Their role in how actors frame their problems and what the output of their reflections look like also needs to be questioned. This is equally important for the researchers conducting vision assessment projects, both as critical observers of vision-oriented communication and as an intervening entity in this communication process.

Cultural semiotics has the task of examining both sign systems in a culture, and cultures as sign systems [33]. Culture as a sign system includes society, described as sign users; civilization, described as a set of texts (not only written ones), and mentality, as a set of conventional codes. By focusing on sign systems, cultural semiotics traces the relations between the three domains [33]. The doubling of the relationship culture–sign system is conditioned by the recursive processes of culture formation: Culture is shaped by signs and as such it is described as a sign system. The cultural semiotic perspective is applied in the following, as we observe that visions of the future are not only means in communication processes; as symbolic products of the social imagination, they play a special role in technology-oriented cultural development. This means that no matter how actors position themselves in relation to technology, and whether they reflect critically on a technology or not, they often do it in relation to modern visions of the future. In this case, actors will continue to strongly tie the logic of modern technological development into their decision-making. This is because visions of the future are strongly intertwined with modern thinking. As social and cultural scientists like Luhmann [34], Kosseleck [35] or Assmann [36] have shown, in modern times there is a disintegration of time into three categories: past, present and future. This is further related to five cultural aspects of modernity: ‘breaking up time’, ‘the fiction of the new beginning’, ‘creative destruction’, ‘the invention of the historical’, and ‘acceleration of change’ [36]. As a consequence, the way of orienting oneself in relation to the future in the modern age is contingent and has an effect on the results of the reflection [37]. This also affects vision assessment projects and its transformative practices.

The question about the role of visions of the future in cultural development corresponds to the general cultural semiotics question about cultural change: How do visions of the future reproduce modern, future-oriented culture? Cultural semiotics asks how it can be that members of a culture both shape this culture and, are simultaneously shaped by the culture. This reciprocal process is conceptualised under the term *cultural mechanism* [33]. It will be shown here that this interrelation can be analysed by applying the concept of *inferences*.^8^

In the following, we refer to the cultural mechanism as the condition of politicisation of concepts appearing in society. Thereby, we focus on what is needed for cultural change to occur. The concept of the cultural mechanism opens the possibility to reflect on the prerequisites and limits of the politicisation process of transformative vision assessment by focusing on culture as an effect of the ongoing communication process. In this way, the concept enables reflection on the framing assumptions in the vision assessment approach, as it offers a meta-reflection on the relation of vision assessment to visions of the future. The process of inference illustrates the way of reasoning in the communication processes: *How do actors perceive and communicate?* According to the semiotician Charles S. Peirce, there are three types of inference, all of which are applied in communication processes: *hypothesis, deduction and induction* [38, 39]. *Hypothesis* (also called abduction), is a vague assumption in the conclusion process, based on common knowledge. *Deduction* is derived from knowledge about the context, in which the linguistic (or e.g. pictorial) concept occurs. The third inference, *induction*, is based on knowledge of concrete sign-like statements. This means that the communication process consists of an interaction between three types of reasoning based on: General knowledge of how to perceive signs (hypothesis), knowledge about the rules of reasoning in the context in which a statement is made (deduction) and how they can be found in the patterns of interpretation, and knowledge about the subject of communication itself (induction) [37]. The inferences both reflect the process of sign use, and can be used to understand and analyse the process of sign use, as we do here. Through inferences one can work out the rules that govern the contexts in which a cultural artefact such as technology is positioned. We will show that the cultural mechanism in the form of mutual influence of the general rules of culture formation, the rules of context, and the new rules that emerge as a consequence of the impact of visions of the future is the condition of the meaning negotiation, e.g. the politicisation of the future. This is a consequence of the openness of signs for interpretation. In order to focus on the mechanism of cultural/societal change, we introduce terms which describe the process of framing and negotiating our problem, as defined in the corresponding debates of RRI (e.g. [5].). These terms are (1) *performance*, (2) *standardisation* of meaning, and (3) *semiotic* *rupture* [37].

1. *Performance* makes it apparent that cultural development is an ongoing process in which existing structures and established rules of social life constantly influence one another. Every rule, every pattern of interpretation, as well as social roles, can be subject to renewed change. In this sense, visions of the future that are the products of an actor or group of actors can again lead to the questioning of the role of these actors, or to the emergence of new actor roles, and the supplement or replacement of existing ones.
2. The tendency to *standardise* meaning describes the tendency to stabilise a social rule. The stabilisation of the rule that leads to the emergence of social patterns makes actors capable of acting. Without a minimum of common understanding and interpretation of the world, communication would not be possible [40].
3. At the same time, it must be possible to interrupt the standardisation process to negotiate the meaning, otherwise there is a risk of losing ourselves in circular reasoning. This can be described as the process of politicisation and re-politicisation, which aims to constantly enable social negotiation according to new recognitions, conditions, and shifts. That is why *semiotic ruptures* play a crucial role in communication processes, and therefore in cultural development. Semiotic ruptures are the moments (statements, realisations, etc.) that disrupt the stabilised meaning, and as consequence, the process of over-standardisation of interpretative patterns, e.g. the process of solidification of discursive patterns.

For this reason, in our analysis, we disclose the process of reasoning and describe the different steps in the reasoning process. These steps consist of: *deductive* *reasoning* (starting from generally valid rules), *inductive* *reasoning* (inferring from individual cases to general rules), and *hypothetical* *reasoning* (inferring from general knowledge about the world and cultural developments). The disclosure of a reasoning process serves to initiate a reflection on the role of vision assessment in each of these steps.

In communication processes, signs are permanently changed due to semiotic ruptures. It is different in the case of symbols (such as societal patterns), i.e. when there is a level of self-evidence in society about the attributed meaning. The meaning of a symbol is well-known and stable, and symbols as such become interpretative patterns. But symbols can also be questioned and thus become the object of negotiation of meaning. In order to include the meaning of a symbol in the process of negotiation, its self-evidence must be *interrupted*. A semiotic rupture occurs, for example, when the interpreter brings a change in context and reveals new sides of the object's being that were not revealed by previous signs.^9^ For example, in a transformative vision assessment, this can be caused by a comparative approach, when two sign codes compete for the role of interpreter in a world segment,^10^ but it can also be caused by context change (cf. the comparative studies proposed by Jasanoff and Kim [41, 42]).

Vision assessment focuses on visions of the future in order to explain their constitutive role for the network in which they operate, as well as for those from which they emerge. The cultural semiotic approach contributes to the question of how the orientation along visions of the future in general influences societal decision-making. In this context, visions of the future can be defined as *cultural techniques* [37, 43]. Understanding visions as cultural techniques means turning to their culture-forming effects. Visions of the future have the following cultural-forming effects: (1) as a product of modernity they contribute to the further development of future-oriented culture. They do this by introducing the past–future distinction. Along the distinction, social communication takes place and decisions are made with regard to whether something belongs to the past or to the future. (2) Visions of the future also introduce further distinctions that arise from their concrete context of action. To give some examples: The vision of “openness” emerged in the context of open source software carries the culture-building distinction open–closed; the vision of “human enhancement”, reflecting the limits of humanity and the relationship between humans and technology, carries the distinction between human and non–human as a culture-building distinction that provides guidance.^11^ Above all, the past-present distinction that underlies visions of the future in general is what guides societal actors today, almost unnoticed, as a framing for the process of negotiating societal development. We assume that although there is sufficient critical reflection on the second type of distinction: e.g. in regard to openness vision, whether openness is enforced and in what way, the first type of reflection on culture-building distinctions of visions of the future, which goes back to the reference to the future that sets limits to the process of decision-making, still receives little attention in the research context on emerging technologies. These distinctions have immense influence on the development of deep codes in society, as they provide a framework that sets the limits of what is imaginable in the culture; respectively what belongs to this culture and what does not [43, 45].

The term *cultural technique* indicates that visions of the future are means of producing culture. In this context, *technique* means a concatenation of actions with power and knowledge that have a culture-forming effect [46]. A vision of the future becomes a node where the actions concentrate [37, 43]. In this way, the cultural semiotic perspective directs the focus of vision assessment to the process of *cultural* *rule formation*, and forces it to question its orientation to the future. The consequence of this orientation is the further development of a future-oriented culture based on the constant discarding of the old in favour of the new [36]. As an effect, everything is calculated on the basis of reference to the future. What does this interrelation imply for transformative vision assessment?

The interventions of transformative vision assessment are close to semiotic ruptures, as they lead to questioning the self-understanding of what emerging technology is or could be, and what it means for society. But still, transformative vision assessment does not reflect the framing process deeply enough. The semiotic perspective on vision assessment has the task to ensure that the framing process of transformation is part of the reflection in transformative vision assessment. The main task is to improve the reflexivity in such a way that the societal changes it intends to foster are not hindered because the vision assessment falls back into old societal patterns of interpretation. The risk is that the logic of modernity, which positions technological development as the origin of societal progress, prevents the emergence of new social rules. As we will show, the hypothetical approach, where the task is to open the space for negotiation, and which searches for new social patterns and/or seeks to break up old ones, can contribute significantly to the transformation of society. Transformative vision assessment is intended to generate such semiotic ruptures in order to initiate a reflexive politicisation process regarding the purpose of technological development in which different parties participate in exchange. But, as we will elaborate, transformative vision assessment does not exploit its full potential in this regard, since it has no terms that support reflection on the framing of the transformation process. This kind of meta-reflection is to be complemented by the cultural semiotic view on visions of the future and technological development.

## Cultural semiotic reflections on prerequisites and limits of transformative vision assessment

In the following, we will show how cultural change is carried out, in order to reflect what is necessary to implement a transformation process. For illustration purposes, we use the project *Vision assessment of scalable 3D printing* (2019–2022). Firstly, we will provide some background information on the accompanying activities in the research cluster 3DMM20. Secondly, this compilation provides insights for cultural semiotic reflection on the prerequisites, limitations and opportunities for improving the transformative vision assessment approach.

### On the transformative process of vision assessment on 3D printing futures

The 3DMM2O cluster consists of around 100 scientists of physics, chemistry, bio- and material sciences from two universities, who conduct research in order to take 3D printing to the “next level”. That is, “to establish scalable digital three-dimensional (3D) Additive Manufacturing reaching all the way from the molecular, via the nanometre and micrometre, to the macroscopic scale”.^12^ Scalable 3D printing should make it possible to “print” very precisely, e.g. functional materials and organic tissue. The R&D aims to overcome “limitations of standard machining, and places the production of materials, objects, and functional devices from the hands of few factory owners into the hands of many with access to tabletop instruments with 3D printing capabilities.”^13^

In general, 3D printing are not new technologies and beyond, 3D printing technologies are actually very diverse. 3D printers have been in industrial use since the 1980s. But there was a visionary euphoria following the invention of the first open source 3D printers at the beginning of the twenty-first century. Visionary hype around 3D printing as a revolution in manufacturing reached its peak around 2015, partly as a result of the printed bust of Barak Obama (2013), which served as an icon in the visionary discourses around 3D printing technologies (see [26]). Subsequently, open source 3D printer developments and the uses of 3D printing have been accompanied by visions of radically new forms of production and consumption of things. The uses of 3D printing technologies have also changed the imaginaries of production and consumption, e.g. based on societal experiments of FabLabs and other maker spaces with open source 3D printers, which aimed to open up the technology for broad societal use. 3D printing has gained increasing mass media attention [8]**.** There are many visions that highlight the potential of the technology, which are widely shared in broad areas of society, such as civil engineering, architecture, health care, and education. Additionally, experimental trials and applications of the technology gained renewed attention in society during the COVID-19 pandemic due to demands for, e.g. 3D printed masks or respirators [47].

The *Vision assessment of scalable 3D printing* project aimed to improve awareness of the societal contexts and needs of future applications of the technology, among both the STEM scientists (science, technology, engineering, and mathematics) within 3DMM2O, as well as relevant stakeholders beyond the cluster. Improving awareness also includes minimising the risk of hype and disappointment, which could be an effect of unrealistic expectations concerning the potential of 3D printing, again both within the cluster and among external stakeholders. The aim of the transformative vision assessment project required a specific methodological design fitting the research context. Dialogues had to be created between society and the STEM research of 3DMM2O in order to open up reflexive space for anticipation. This space was needed to identify the societal and technical demands of change, and this was needed to provide orientation for developing 3D printing technologies of the future as a contribution to urgent societal changes [26]. To enable this, the vision assessment project had to enrich the technology-driven vision of the research cluster.

*Vision assessment of scalable 3D printing* identified six key narratives in the discourses surrounding 3D printing:

1. “Shaping the world atom by atom and bit by bit”
2. “Communal Empowerment”.
3. “Individual Empowerment”.
4. “The next industrial revolution”.
5. “Enhancement of human health”.
6. “Eco-efficiency”.

These six narratives revealed a wide range of heterogeneous visions—the most prominent concerning hopes regarding the future potential of 3D printing in the 2010s [8, 26].^14^ All these narratives imagine overcoming a specific challenge of the present—but in contrasting directions. This becomes apparent when we juxtapose, for example, the narratives of “eco-efficiency”, which aims at deceleration, and “next industrial revolution”, which aims at acceleration. Further in the vision assessment research process the juxtaposed key narratives were used *to provide orientation and facilitate reflexivity* in the communicative exchange with scientific experts in 3DMM2O and with external societal stakeholders. In their function as means of communication, the key narratives were used in diverse inter- and transdisciplinary feedback-dialogues. These dialogues were used to open the participants’ imagination and to shift the focus from their context-specific view on 3D printing technologies (i.e. nanolaser-printing in the lab, use of 3D printing in a factory, 3D printing in maker spaces/FabLabs) to the wider contexts of society. During the feedback-dialogues, it became evident that the most important values underlying reflection, development, and use of 3D printing in a responsible way are *sustainability* and *inclusion* [26].

The key narratives served to *enable* communication about the different values which are and could be attached to 3D printing technologies, especially with the focus on sustainability and inclusiveness. As means for enabling communication, the key narratives served as a starting point for improving reflexivity. The task of *Vision assessment of scalable 3D printing* was to turn the way these narratives are guiding debates about 3D printing, from asking, Which societal problems will be solved by certain 3D printing technologies?, to What has to change in society so that the technology could be used to solve societal problems? This was done through a participatory and co-creative scenario process, in which the participating scientists and societal stakeholders anticipated potential 3D applications in contexts such as social inclusiveness and sustainability [26]. The scenarios served as means to enable a reflexive process of deliberation to think about the futures of 3D printing technologies in society *differently* compared to the technology-driven visions of the key narratives*.*

Semiotic ruptures about what the technology is being developed for can be introduced in this way in the development process of a technology. The cultural semiotic perspective applied below shows how deeply the transformative vision assessment project has intervened into cultural structures at this point and reflects on what is further needed for transformation processes to be conducted.

### The cultural-semiotic interpretation of vision assessment of scalable 3D printing project results

In the following, we reflect on the involvement of the transformative practice of vision assessment in the process of cultural change, and what is needed for societal transformation to occur. This section exemplifies the role of vision assessment in the transformative process and discusses its potential, in order to find out to what degree a project of transformative vision assessment such as *Vision assessment of scalable 3D printing* currently contributes to societal transformation, and to reflect on how transformative practices can be further improved. The three steps in reasoning—hypothesis, deduction and induction—representing the cultural mechanism, will be applied. This shows which transformative steps the vision assessment already considers and where there is room for improvement.

#### General rules of culture formation

- Opening the space of reflection

The transformative practices of vision assessment aim to co-shape and modulate the way society negotiates the goals of technological development. In order to assume the position of a reflexive and modulating authority, vision assessment is challenged to act at all levels of transformation and to offer a meta-reflection on this process. The hypothetical reasoning introduced below will show how transformative vision assessment can reflect on its own practices, and why this is needed.

Hypothesis is the most creative reasoning step, and according to Assmann [44], is the one used by artists and the humanities. Its task is to open the space of reflection. Hypothesis allows us to move beyond the known patterns of interpretation and thus to increase creativity. Assmann calls it the wild semiosis. “Wild” is the semiosis because it does not follow social patterns^15^; instead it looks beyond the framework of the known interpretative pattern and opens room for imagination.

Following the rule of performance (see “Cultural semiotic approach to Transformative Vision Assessment” section), it can be assumed that there is a process of negotiating the meaning about every newly discovered segment of the world, such as a new technology. In addition, any sign shows a tendency towards standardisation of meaning, and further to over-standardisation [33]. In this way, the sign attains a central position and produces patterns of interpretation in society. Patterns of interpretation are deep structures that are so self-evident that one no longer notices them. Rather, one perceives the world through their “lens”.

One such “lens” of modernity is provided by visions of the future. They frame the process of negotiating how a society wants to live, and relate decision-making to the future, and thus to the need for progress and constant discarding of the old in favour of the new. The hypothetical conclusion has the task of questioning these very basic patterns of interpretation of modernity. Since the reference to the future, which in modernity is strongly related to technological development, affects the process of negotiation in society, in the process of transformation toward RRI this way of framing the process should also be questionable. This can be seen, for example, in calls for deceleration and sufficiency [48] which are contrary to modern innovation logic, or in warnings about consequences of the future reference, which result in postponing decision-making [49]. It is at this point that we follow up on the calls from RRI [5] to reflect on the process of participation, not only as a process where policy issues are discussed, but also where the framing process is reflected. This questioning requires the conscious introduction of semiotic ruptures in R&D that would lead to the breakdown of existing reasoning patterns.

b) Hypothetical reasoning as a step in the transformative vision assessment process

Following the rules of the cultural mechanism, it can be assumed that with the emergence of 3D printing technologies, a process of negotiation about what the technologies are and can become will occur. In this process, transformative practice can start by showing the interpretative patterns that are used to frame the technologies (as it already does), and then reflect on the transformation process itself. Thereby, transformative vision assessment needs to (1) question the existing framing of the negotiation process and provide a meta-reflection, (2) look at the existing cultural patterns that the transformation praxis follows currently, (3) reflect on the potential of the technology itself. Points 2 and 3 will be verified in sections “Rules of orientation towards the future “ and “Emerging rules in the context of 3D printing technologies “. In the following, we present the assumptions about opening the reflection on the basis of the hypothetical approach.

The observation of the emergence of 3D printing in society within *Vision assessment of scalable 3D printing* has shown that there are different tendencies in the development of 3D printing. In societal debates, 3D printing is connected to the sustainability discourse, but as new very flexible technologies they can also support the further development of unsustainable practices, such as producing small parts which are quickly discarded and replaced. The scenario development in the *Vision assessment of scalable 3D printing* project has shown that it is not self-evident that 3D printing will lead to sustainable development, and that in order to implement this shift towards sustainability, values such as inclusivity must also be implemented and promoted [47]. The scenario process had the goal of opening reflection on what is needed for cultural change to occur, and only then was it considered how the technology can support this process. Thus, the logic of development was reversed, so that one tries to escape technology determinism.

In the process of transformation, reflection on the framing process must be ensured. As we can see, existing methods can support the questioning of technological determinism. But it should also be possible to question the process of societal negotiation, by creating space for conscious introduction of semiotic ruptures and the conscious handling of and reflection on symbolisation processes (including performance and the tendency to standardisation). The semiotic ruptures must also serve to reflect the reference to the future and the consequences of this kind of orientation. How such ruptures can be introduced, can be observed by art^16^ (see also [50]). One of the tasks of art (depending on the art discipline or artist) is to confront the recipients with existing entrenched patterns of interpretation and thus offer space for rethinking. Possibilities to apply the hypothetical approach by reflecting ruptures could include (a) context change, (b) “misuse” of objects for completely different purposes than intended, (c) change of interpretation patterns that say something about the purposes of development in general, (d) thinking in spaces, i.e. a game where, instead of time-related visions of the future, spatial utopian worlds alternate with modern utopias to work out the differences, (e) playing with randomness by not arranging or assigning things according to existing orders. This can support the questioning of the framing process and avoid a situation where despite the will to escape technological determinism and the modern logic of progress, the interpretative patterns of the latter will continue to be applied to R&D.

#### Rules of orientation towards the future

- Searching for interpretative patterns

The next step in reasoning is deduction, which is about looking for established patterns of interpretation in society. The deductive reasoning introduced below shows what kind of already existing interpretative patterns the transformative vision assessment needs to reflect, and what patterns of interpretation it is itself involved in. Deduction goes back to the conventionality of signs. Conventionality means that signs are based on agreements, or habits of a speech community. These are represented by rules for reasoning. In deduction, the rules of the concrete context are applied. The context of modernity and its rules of focus on time and progress [43], which is visible on the orientation along visions of the future, is also to be considered here.

b) Deductive reasoning as a step in the transformative vision assessment process

The context of modernity includes the rule of orientation and communication by reference to the future. Visions of the future not only contribute to orientation, but by focusing on the future they also contribute, e.g. to the modern tendencies of discarding the old in favour of the new. These characteristics of modernity can be seen as a consequence of the following rule: To develop society further, the innovation process needs to be driven forward, and it must be focused on the future and constantly developing new ideas. What consequences the reference to the future has, and whether this serves to keep the process going instead of providing solutions here and now [49] must therefore be reflected, also in the context of the intervention of transformative vision assessment along visions of the future (see “Cultural semiotic approach to Transformative Vision Assessment “ section).

Consequently, in the orientation towards the future, ideas and concepts are discarded even before they are realised. This was observed in the context of 3D printing. The great interest in 3D printing leads to the emergence of many start-ups. Grassroots innovations, i.e. community-led solutions, emerge quickly and locally and many prototypes are developed. This leads to increasingly less focus on concrete products and ever more on ideas (see also [51, 52]). This is a consequence of the rule of acceleration in modernity, which requires R&D to increase.

The orientation towards the future in technology development results in organisational forms, actor roles, and emerging artefacts being measured by their future value. The focus is what is considered necessary for future development, and what is considered part of the past will be discarded. However, since it is not clear what may or may not be of importance in the future, what is seen as necessary depends on the general design today of the desired future. That is why the participation process, as in the scenario process in transformative vision assessment projects, *should* involve different stakeholder groups also to question the design of these processes, beside reflection on the policy issue itself. Reference to the future can easily be questioned when different stakeholders are involved in the participation process, for it can be assumed that not all parts of society will submit to the type of orientation prevailing in modernity, i.e. orientation along the future. Rather, the participating stakeholders are to be asked in what ways the future plays a role for them when they cope with problems, and in what ways they orient themselves differently.^17^

#### Emerging rules in the context of 3D printing technologies

- Separating the aspects of the technology with the potential for change

The next step in reasoning, induction, represents the new rules which emerge from the concrete case and lead to the emergence of interpretative patterns. Here, the transformative vision assessment can focus on: What does the concrete technology change in the culture in which it occurs? What are the societal tendencies which it supports? With this step in reasoning, vision assessment can work out what the new object (a technology) currently enables and what it could enable in society. In this way, we catch the aspects of the technology with the potential for change and separate these from the prevailing social patterns (which were reflected in the deduction reasoning).

b) Inductive reasoning as a step in the transformative vision assessment process

Applying this step of reasoning, we need to focus on the object itself, here the 3D printing technologies, abstracting from its current context as much as possible. We need to analyse the characteristics and aspects of the object and their potential for change. In order to work out the special characteristics of the particular technology, vision assessment can also observe how the different interpretations in the form of visions of the future associated with 3D printing influence one another, and how their *joint performance* affects other elements of the network. We ask: Do their patterns of argumentation and assumptions intermingle? What does 3D printing mean, depending on which vision is attributed to it? This is already part of the scenario process, but it must be emphasised more strongly and abstracted from the existing patterns of interpretation, with all its aspects and characteristics. This practice would be similar to an ethnographic study, where from the intensive observation of an object its application and context are reflected. We also consider that separate recording of these properties in the form of documentation is important, as in this way, the prevailing societal rules with their patterns of interpretation can be played off against the properties of the technology in the participation process, and considered separately.

To abstract from the contexts in which 3D printing arises, a tested method is to compare and switch contexts. The results of the *Vision assessment of scalable 3D printing* project show that different visions of the future are applied to 3D printing for the purposes of interpreting the technology. This way, 3D printing is interpreted through existing interpretation patterns in society. The next step is to elaborate whether and how far the impact of the concrete technology produces new patterns of interpretation, or does it submit to the prevailing social rules?

The visions of the future surrounding 3D printing development represent current societal challenges, such as the demands for progress, inclusivity, individual empowerment, acknowledgement of local needs in contrast to global needs, the urgency of climate change, and ethical questions about what make us human and how we can offer better life conditions to broader parts of the world. In reality, these questions do not stand alone and are interrelated by tradition. The visions regarding communal empowerment and sustainable development are brought together in different contexts, and ever-increasingly as bottom-up solutions for climate change. However, it is partly due to the open source 3D printer that these visions became connected to one another. In the spirit of “print the world the way you want”, the open source 3D printer, which is open to wide parts of society and allows individual production, answers to the next step of the 4th Industrial Revolution with its demand for more accessible and suitable products. With the upcoming urgency of action in the context of climate change, the purpose of individual and local production is reframed and connected to sustainability goals.

In the case of 3D printing, the mutual influence of at least *three visions* can be observed: The reference to ever smaller, more accessible and more powerful 3D printers is a reminder of the scientific goal to develop “finer, faster, and more”. It is connected to the vision of “shaping the world atom by atom and bit by bit”. Furthermore, the reference to cheaper and more accessible 3D printers suggests that the goals of developing technologies for manufacturing should be redefined, and opens up the possibility of imagining the 4th Industrial Revolution as one that relates to local and individual production. The latter is connected to the goals of sustainability, although not in every societal area. As a consequence, a discourse emerges that leaves room for a variety of interpretations of how these three visions are connected to one another.

3D printing can be interpreted in different ways. As has been shown in the scenario process of the *Vision assessment of scalable 3D printing* project, even individual and local production is not necessarily connected to the application of the 3D printer. A development in which mass production predominates is both conceivable and possible. The industry is already implementing 3D printing technologies for industrial production (e.g. healthcare, aerospace, military, transportation, motorsports). So, even if tendencies towards societal challenges such as individualisation and decentralisation are present and desired, they do not have to be automatically related to the goals of sustainability and inclusivity in the 3D printing context. Rather, the discourse first involves a negotiation of how the challenges presented by the visions are connected and which of them is the most significant to focus on. For example, with a focus on technology progress, the next steps may fail to meet the goals of local and/or sustainable production. If technology progress is one of the most important rules in society (see “Rules of orientation towards the future” section), then the desired tendencies towards inclusivity (see “General rules of culture formation” section) and sustainability will be measured by their usefulness for technology development. So, it can be said that 3D printing becomes a node that brings together the three societal challenges. From the interpretation of the connection of the visions to one another, cultural rules can be identified. It is the task of transformative vision assessment to analyse and observe if the rules meet the societal demand, and whether they are actually exploiting the potential of the technology to meet societal change.

The *modulation through co-creating sociotechnical scenarios* by this transformative vision assessment project is opening up space for reflection. The confrontation of the individual actors with the unfamiliar interpretations of development goals can lead to semiotic ruptures. The scenario method in vision assessment is used for this very purpose: To interrupt the self-evidence of the goals of technology development. This is further complicated by the fact that, as shown in “Rules of orientation towards the future” section, visions of the future along which society orients itself, which vision assessment uses to reflect on current societal developments, are part of the social system to be reflected. If visions of the future support the rules of modernity, then there is a possibility that they will eventually lead to the support of this order. The first step is taken with the scenario method in the vision assessment: To turn the reflection process, first asking about general socially desired values and societal challenges, and then about technologies. Further, as we argue here, the vision assessment must also be able to think beyond the technological vision of the future by (1) questioning the existing patterns, as described in “General rules of culture formation” section through, e.g. context change, “misuse” of objects for different purposes than intended etc.; (2) discussing and reflecting on the design of the participation process, as described in “Rules of orientation towards the future” and “Emerging rules in the context of 3D printing technologies” section. Finally turning to the possibilities offered by the technology itself, abstracting as much as possible from the contexts in which the technology occurs, as described in this section. The entire process would be based on the attempt to view the object “3D printing technologies” without prejudice, i.e. independent of existing patterns of interpretation in society. Since it is not actually possible to completely abstract from patterns of interpretation, the three steps of reasoning (hypothesis, deduction and induction) can be applied to look at 3D printing from as many perspectives and different assumptions as possible, thus abstracting from a single interpretation. These steps in reasoning are not to be arranged sequentially, but are constantly weighed against one another. They also serve, as already mentioned, to abstract various aspects of the process of assimilating technology into social structures, such as the general ability of the actors to interpret and create new patterns of interpretation (“General rules of culture formation” section), the disclosure of the prevailing patterns of interpretation, in which the vision assessment is embedded (“Rules of orientation towards the future” section), and turning to the technology itself in order to reflect on its potential, abstracting as far as possible from the patterns of interpretation.

Transformative vision assessment faces the challenge of co-shaping the current demands for cultural change by rigorously reflecting on current societal challenges, and questioning the logic of visions of the future, which also provide orientation in vision assessment. As a consequence, inclusivity and sustainability as values are reflected and evaluated by different stakeholders, including regarding aspects of modernity, through the intervention of vision assessment.

## Conclusion and discussion

Currently, there is increasing demand for change in the values underlying today's culture, as for example, when UNESCO calls for “a Culture for Sustainable Development”.^18^ Governmental as well as non-governmental organisations increasingly point out that in order to achieve sustainability goals and to improve the standard of living of the world’s population, social change must occur. To achieve such change, however, the values and beliefs that underlie the culture must be confronted with current challenges and re-examined. As research has already shown, the visions of a 4th Industrial Revolution or local production are not leading to sustainability or to inclusivity per se (see [26]); they rather open up space for negotiations about the future we want to live in. So, first of all, they are subject to politicisation, as the key narratives identified in the *Vision assessment of scalable 3D printing* project have shown.

The scope of politicisation into cultural development can be observed in the set of visions of the future, which offer different interpretations of research and innovation. Such visions of the future serve as orientation in decision-making. From the cultural semiotic perspective, the cultural mechanism with the three steps of reasoning—hypothesis, deduction and induction—in which visions of the future are involved, has an impact on the formation and development of deep cultural structures. In parallel, the cultural mechanism reproduces existing cultural structures, including patterns of interpretation. To ensure that the negotiations about the interpretation of the meaning of technology lead to desired social and cultural changes rather than being subject to an interpretation that allows one stakeholder to use the technology for its own purposes without regard to socially desired changes such as sustainability, there must be space for questioning the interpretation frames and for re-politicisation, i.e. for re-negotiation of the purpose of the technology and the societal patterns of interpretation.

The cultural semiotic perspective shows prerequisites of the politicisation of the future, and explains how it can be that visions of the future become props of politicisation and at the same time are subject to change. This is because visions of the future are both a part of the culture in which they emerge and reproduce existing rules of modernity, whilst they also produce new societal patterns of interpretation by interconnecting different societal contexts. We can see this as 3D printing becomes a node between three visions of the future (4th Industrial Revolution, inclusive and local production, sustainable production and consumption). For transformative practices such as in vision assessment, this means that the process of framing the transformative practices needs to be reflected as well. In the case of vision assessment, the orientation along visions of the future is to be questioned, and its cultural-building function, as well as its possible consequences, need to be discussed. Can these visions be also a kind of obstacle to the transformation processes, as they follow the same logic of progress as the technologies? Further, is it possible to modulate the visions in such a way that allow meta-reflection? We argue that it is possible, and further that a hypothetical approach which opens reflection to development of new rules in society by involving different stakeholders in the discussion of the way the participation process is designed, could support it. Lessons from arts and humanities can be learned on how to break with existing societal patterns and open the space for the *re-politicisation* of how societal problems frame societal discussions today.

This kind of meta-reflection is needed to escape the risk that vision assessment, by orienting itself along visions of the future in its transformative practice, becomes just another actor in the politicisation process. In order not to get caught up in the constraints of the reference to the future and fall into values such as acceleration, or constantly throwing away the old in favor of the new, vision assessment is required to reflect on the reference to the future (its own as well as that of the other actors). The role of vision assessment in the politicisation process is unavoidable; however, it can be mitigated by meta-reflection on its own orientation to the future, and related visions of the future. In this way, vision assessment would ensure that its transformative practices become a kind of governance for ensuring the acceptability, desirability and sustainability of development.

The role of the co-creative scenario process in *Vision assessment of scalable 3D printing* was to support R&D by triggering the process of reflection on the purpose of the technology and the underlying values. This was achieved by changing the perspective; not starting the discussion about the technology from the point of view of technological development, but by first debating socially desired changes and their underlying values and then, in a next step, discussing the use of the technology to achieve the goals. In this way, semiotic ruptures can be created and a negotiation about technology can be initiated.

By considering visions of the future as a product of modernity, a limitation of transformative vision assessment becomes apparent. Even if TA is oriented towards possible futures instead of the future as constructed, with alternative scenarios for anticipatory negotiations (i.e. [2].), and turns away from the past–future dichotomy, it must always reflect its reference to the future as a point of orientation. Transformative vision assessment must interrogate itself about which developments it is supporting through an orientation towards the future.

The cultural semiotic reflection illustrates how 3D printing becomes subject to negotiation in the society. In this way, it is negotiated by asking through which patterns of interpretation existing in society, are the purpose and use of technology to be interpreted. It also shows which patterns of interpretation are already reflected in the vision assessment and which are not. A rupture and cultural change can also occur here when 3D printing becomes a node which brings together different visions and thus also contexts, resulting in a new constellation. The open source 3D printer is not only generating hype around the technology, but is also helping collective thinking about at least three societal challenges—inclusion, sustainability, and economic production. We can therefore see 3D printing becoming a node between three visions of the future. The open source 3D printer has a strong discursive impact because it is at the same time a visualisation of the 4th Industrial Revolution of sustainable and individual production, and a concrete technological solution that can contribute to solving some problems of climate change, reducing global or state-led overproduction of goods. In this way, it has caused a semiotic rupture which has led to a re-thinking of values in society. But, as observed in the *Vision assessment of scalable 3D printing* project in the Cluster 3DMM2O, change is happening slowly and is far from affecting all areas of societal life. The question arises whether the R&D in the cluster can achieve its goals of major change in society through the use of technology towards sustainable, inclusive, human-friendly production, or whether technology will address the current issues of the existing social order with consequences of further climate change, social injustice, monopolisation, etc.

Cultural semiotics is concerned with the role of signs and sign systems, including language, images and formulas, in cultural development. In this paper, it serves to build a theoretical foundation to link linguistics, literary studies and media studies to research in TA. These sciences can be understood not only as supporting disciplines, but can also be used to answer fundamental questions about the way technologies are currently discussed, the influence technologies have, including on *cultural development*, and about how those negotiations can be modulated differently.

## Data Availability

Not applicable.

## Footnotes

1 https://www.3dmattermadetoorder.kit.edu/

2 https://www.itas.kit.edu/english/projects_loes19_va3d.php

3 Open source is a term that originates from open source software. Open source is code (also hardware or idea) that is available to the public; it means that anyone can view, modify and distribute it.

4 On the on the debates in visionary literature on 3D printing and the critical analyses of the visionary promises in STS concerning these challenges see, e.g. [9–15]

5 By sign-like concepts we mean, e.g. linguistic, pictorial or other statements.

6 This is based on the insights of various vision assessment studies over more than a decade, and the results of related STS studies on how sociotechnical futures are shaping processes in the present (e.g. [16]).

7 This lack is addressed, e.g. in [26]

8 Inference is a term of logic and is defined as a step in reasoning, e.g. moving from premises to logical consequences. In our theoretical approach we fall back on the theory of sign of the semiotician and logician Charles S. Peirce [38] when we talk about inferences.

9 Such semiotic ruptures are actively applied by artists to open up reflexive space, as in the context of ready-made artworks, when everyday objects are placed in museums to open up a space for reflection on their purpose, and thus on the people using them and the culture out of which they emerge, see for example the work Fountain by Marcel Duchamp.

10 The clash of visions described in [1] could be seen as an example of this.

11 This orientation serves to make sense of the world and is the basis of cultural formation ([43], see also [30]). Cultures arise from the need to make the world readable, i.e. interpretable [44] from the attempt to give meaning.

12 https://www.3dmattermadetoorder.kit.edu/vision.php

13 https://www.3dmattermadetoorder.kit.edu/about.php

14 The visionary key narratives can be found in the visionary literature on 3D printing futures (e.g. [9, 11, 14]) and are deconstructed in the observing STS literature [10, 12, 13, 15].

15 Besides artists, eccentrics or madmen are those who know how to implement wild semiosis [44].

16 The interdisciplinary field of art as research offers many reflections on how the artistic approach can be applied to scientific knowledge.

17 For example, the spatial kind of orientation known from myth is still to be regarded as a competitor to the modern temporal orientation (for the comparative study between myths and visions of the future, see [37]).

18 https://en.unesco.org/culture-development
